# Four lab tests to predict intrauterine insemination outcome

**DOI:** 10.4103/0974-1208.57232

**Published:** 2009

**Authors:** Viroj Wiwanitkit

**Affiliations:** Wiwanitkit House, Bangkhae, Bangkok - 10160, Thailand

Sir,

I read the current Prabhu *et al*. publication, on the possibility of usage of four laboratory analysis plasma protein thiols, ceruloplasmin, C-reactive protein and red blood cell acetylcholinesterase to predict the outcome of intrauterine insemination (IUI), with great interest.[1] To set up the clinical usefulness of the four laboratory tests for this situation, proposed cut off values with data from ROC curve analysis need to be given. Indeed, all four laboratory parameters are considered non specific and the observed trend in the published work might be due to other factors, especially for the pre-analytical factors; but these items have to be further addressed to help judge and include the four tests for prediction of IUI outcome.
